# Enhancement of gemcitabine toxicity and specificity through PI3K/Akt/Nrf2 pathway inhibition in pancreatic cancer

**DOI:** 10.3389/fphar.2026.1724989

**Published:** 2026-02-16

**Authors:** Yu-Shan Chen, Stephen O’Hagan, Philip J. R. Day

**Affiliations:** 1 Division of Evolution, Infection and Genomic Sciences, Faculty of Biology, Medicine and Health, The University of Manchester, Manchester, United Kingdom; 2 School of Chemistry, Department of Chemistry, Dover Street Building, The University of Manchester, Manchester, United Kingdom; 3 Department of Medicine, University of Cape Town, Cape Town, South Africa

**Keywords:** binary drug, chemoresistance, drug sensitiser, enhanced drug efficacy, PDAC, PI3K/Akt/Nrf2 pathway

## Abstract

**Introduction:**

Pancreatic ductal adenocarcinoma (PDAC) is a lethal malignancy associated with rapid metastasis and chemoresistance driven by PI3K/Akt/Nrf2 signalling and drug efflux transporters. The lack of symptoms and early diagnosis are clinically challenging, and the development of new medications is limited. Therefore, a new strategy to enhance gemcitabine efficacy without increasing systemic toxicity has been demonstrated.

**Methods:**

The fragment-based drug sensitiser BD B10 was selected from a Maybridge fragment library using the Tanimoto coefficient to identify structural similarity to trigonelline and tryptamine. PDAC cell lines and non-cancerous pancreatic cells were reated with gemcitabine, BD B10, or their combination. Cell viability, apoptosis, migration, and signalling pathways were analysed using microscopy, flow cytometry, RT-qPCR, Western blot, and RNA Seq with pathway analysis.

**Results:**

Applying BD B10 in PDAC cell lines reduced the dose requirement of gemcitabine by 10%, with no adverse effects on growth of non-cancerous pancreatic cell lines, enhancing drug efficacy by 12%, with a otential marked gain in therapeutic index. Additionally, combination treatment enhanced apoptosis, reduced migration, and impeding PI3K/Akt/Nrf2, STAT3, and Wnt/β-catenin signalling regulation.

**Discussion:**

BD B10 was identified as a non-toxic drug sensitiser that enhanced gemcitabine efficacy in PDAC cells and improved the therapeutic index by inhibiting key survival and resistance pathways. Specific roles for BD B10 in PDAC were identified and further testing may prove drug sensitisers have a more general application to enhance drug therapies.

## Introduction

Pancreatic cancer is ranked the 3rd leading cause of cancer-related mortality in the United States and 7th worldwide, with approximately 90% of cases being pancreatic ductal adenocarcinoma (PDAC) (Stoffel et al., 2023; Partyka et al., 2023). Since PDAC lacks early symptoms and has a late diagnosis, PDAC is predicted to surpass colorectal cancer as the second leading cause of cancer death by 2040 (Rahib et al., 2021). With early metastasis and drug resistance, plus rapid tumour proliferation, pancreatic cancer has become one of the most lethal malignancies, with a 5-year survival rate of around 12% (Springfeld et al., 2023; Halbrook et al., 2023).

The usual therapeutic approach for pancreatic cancer includes chemotherapy, targeted therapy, radiotherapy, and surgery (Stoop et al., 2024; Zhao and Liu, 2020). Chemotherapy remains the most common treatment, especially in advanced pancreatic cancer with distant metastasis: 80%–85% of patients with PDAC are diagnosed at a late stage, making them ineligible for surgery (Li et al., 2023; Michel et al., 2022).

Gemcitabine is widely used as a chemotherapeutic agent to treat pancreatic cancer patients (Moysan et al., 2013). Nevertheless, its efficacy is limited due to the promotion of rapid multidrug resistance (MDR) through metabolic reprogramming (Nong et al., 2020; Shigeta et al., 2023), tumour stemness, and drug efflux by ATP-binding cassette (ABC) family members, including MDR-1 (ABCB1), MDR-associated proteins (MRPs/ABCCs), and BCRP (ABCG2) (Marin et al., 2022; Adamska and Falasca, 2018). This resistance is mediated through the activation of PI3K/Akt/Nrf2 and Wnt/ß-catenin cooperating with CREB signalling pathways, facilitating drug efflux and epithelial-mesenchymal transition (EMT) (Huang Y. H. et al., 2024; Barrera et al., 2021). Therefore, overcoming or reversing chemoresistance is an urgent obligatory requirement for the treatment of PDAC.

The aberrant activation of PI3K/Akt/Nrf2 signalling pathway is pivotal in regulating MDR by directly inducing the expression of efflux transporters (Yao et al., 2016; Liu et al., 2020), leading to tumour relapse and cancer progression (Choi et al., 2021). Additionally, the downstream target VEGF promotes tumour angiogenesis, reducing chemotherapeutic drug penetration through reduced capillary permeability (Chekhonin et al., 2013; Gremonprez et al., 2015). Akt inhibitors such as MK-2206 and BAY1125976 (Coleman et al., 2021) are administered to disrupt the tumour-promoting environment. However, current drugs and inhibitors can cause cytotoxicity and adverse effects, such as hepatotoxicity and pneumonitis, which limit their clinical use (Jonasch et al., 2017; Zhang et al., 2019).

Dysregulation of the PI3K/Akt pathway also results in sustained activation of Wnt/ß-catenin cooperating CREB signalling (Liu et al., 2020), promoting EMT and pancreatic cancer progression through aberrant stimulation of Wnt ligands or Frizzled receptors (Javadinia et al., 2019; Kuo et al., 2019), extruding chemotherapeutic agents, thereby diminishing apoptosis and fostering chemoresistance in pancreatic cancer cells (Cui et al., 2012).

Current strategies to combat chemoresistance include combination therapy, targeted drug therapy, immunotherapeutic strategies, and drug repurposing (Ramos et al., 2021). However, limited drug efficacy and toxicity remain significant challenges in clinical applications. A promising new approach involving using natural products (NPs) such as alkaloids, flavonoids, phenylpropanoids, terpenoids, or ABC inhibitors to combat resistant cancers has gained substantial attention for their pharmacological benefits in reversing MDR (Atanasov et al., 2021; Yang et al., 2022; Chen T. et al., 2024). Despite these advantages, NPs face limitations due to complex bioactivities, intricate regulatory mechanisms, poor stability and solubility, and potential adverse events (Huang L. et al., 2024). Additionally, efflux transporter inhibitors may lead to inherited side effects and unexpected drug-drug interactions (DDIs) (Choi and Yu, 2014).

Given the complexity of structures and functions of most NPs, screening chemical fragment libraries is less time-consuming and offer a diverse range of chemical diversity (Shaivi, 2023). The Maybridge rule of 3 fragment (MBF) library was developed for fragment-based drug screening. Our previous study applied the Tanimoto coefficient (Tc) analysis to the MBF library to identify best-fit MBFs that whilst being non-toxic, when used in conjunction with gemcitabine produced an enhanced killing of pancreatic cancer cells. The fragment-based drug sensitisation strategy we previously termed a binary drug (BD) (Grixti et al., 2017).

In this study, we aim to demonstrate the BD B10 to possess a PI3K/Akt inhibitory function and low toxicity, enhancing drug efficacy to overcome MDR and identified the associated regulatory mechanisms in pancreatic cancer.

## Materials and methods

### Drugs and reagents

Gemcitabine hydrochloride (FCB108986, Fluorochem) and desipramine hydrochloride (D3900, Sigma) were suspended at 100 mM and 1000 mM in ddH_2_O respectively, and stored at −20 °C. Tryptamine (193747, Sigma) and N-Methyl-(5-pyrid-3-ylthien-2-yl) methylamine, (BD B10, CC66846DA, Thermo Fisher Scientific) were suspended at 1500 mM in DMSO and stored at −20 °C.

### Tanimoto fingerprint-based similarity analysis

The fingerprint-based similarity analysis was performed by using the Tc to measure the similarity between target molecules. The analysis details were published in our previous papers (O’Hagan and Kell, 2015a; O’Hagan and Kell, 2015b; O'Hagan and Kell, 2015; O′ Hagan et al., 2015; O'Hagan and Kell, 2016) by using the KNIME workflow. The MBF library (Thermo Fisher Scientific) following the rule of 3 was applied to select the most appropriate BD.

### Cell culture

Human pancreatic ductal epithelial carcinoma cell lines: Panc1 possessing *KRAS*, *TP53*, *CDKN2A*, and *SMAD4* mutations and MIA-PaCa-2 (MP2), possessing *KRAS*, *TP53*, and *CDKN2A* mutations were maintained under culture with Dulbecco’s Modified Eagle’s Medium (DMEM) with high glucose (D6429, Sigma) supplemented with 10% heat-inactivated foetal bovine serum (FBS, 10500064, Gibco) and 1% penicillin-streptomycin (PS, P0781, Sigma). Human pancreatic ductal adenocarcinoma cell line BxPC3 that carries *TP53*, *CDKN2A*, and *SMAD4* mutations was cultured in RPMI medium 1640 (11875-093, Gibco) containing 10% FBS and 1% PS. The immortal human pancreatic duct epithelial pancreatic cell line hPDE was cultured in keratinocyte SFM (1X) medium (17005-034, Gibco) supplemented with human recombinant epidermal growth factor (rEGF), bovine pituitary extract (BPE) and 1% PS. Cell lines were routinely maintained at 37 °C in a humidified 5% CO_2_ atmosphere. Panc1 and MP2 were passaged every 3–4 days (sub-cultivation ratio 1:3) using 0.05% trypsin and 0.02% EDTA (T4174, Sigma) for 5 min to detach. BxPC3 was passaged every 2–3 days (sub-cultivation ratio 1:5) using 0.25% trypsin and 0.1% EDTA for 15 min to detach. The hPDE cell line was passaged every 3–4 days (sub-cultivation ratio 1:4) using 0.5% trypsin and 0.2% EDTA for 15 min to detach. The cell density was maintained in the range between 1 × 10^5^ and 1 × 10^6^ cells. mL^−1^ in a 100 mm TC-treated Petri dish.

### Co-administration with gemcitabine and BD B10

Gemcitabine was set up with 7 different concentrations (0.003, 0.01 0.03, 0.1, 0.3, 1, and 3 µM) and BD B10 for 3 different concentrations (3, 10, and 30 µM) for further treatment. Each concentration of BD B10 was with 5000 times dilution (0.02%) to avoid DMSO toxicity.

Panc1 and hPDE cells were seeded in a 96-well plate at a density of 5000 cells/per 96-well, and MP2 and BxPC3 cells were seeded at a density of 10,000 cells/per 96-well. The cells were treated with various concentrations of BD B10 (3–30 µM) for 24 h at 37 °C. After incubation, cells were further co-administered with gemcitabine (0.003–3 µM) and BD B10 (3–30 µM) for either 96 h (Panc1, hPDE) or 72 h (MP2, BxPC3).

### Co-administration with gemcitabine and tryptamine

The tryptamine study was initiated by incubating Panc1 cells with 10 µM tryptamine for 24 h at 37 °C. The following day, the culture medium was replaced with fresh medium containing 0.1 µM gemcitabine and 10 µM tryptamine, and incubation continued for 96 h. The WST-1 assay was performed at the end of treatment.

### Receptor binding competition test

The tryptamine/BD B10 competition protocol was conducted using various combinations of tryptamine (0–10 µM) and BD B10 (0–10 µM) at different concentrations in Panc1 cells for 24 h. The cells were then further co-administrated with 0.1 µM gemcitabine and different combinations of tryptamine and BD B10 for 96 h. The WST-1 assay was performed to assess cell viability.

### Measurement of SERT uptake

Desipramine hydrochloride is an inhibitor blocking the functions of SERT, norepinephrinetransporter (NET), and dopamine transporter (DAT). The inhibitor experiment proceeded by incubating 1 µM desipramine with 10 µM BD B10 for 24 h. After the incubation, cells were further co-administrated with 1 µM desipramine, 0.1 µM gemcitabine, and 10 µM BD B10 for 96 h. The WST-1 assay was conducted at the end of the treatments.

### Cell proliferation/viability assay

At the endpoint of each treatment, WST-1 (ab155902, Abcam) and MTT assay (B7777-APE, APE x BIO) were applied to analyse the cell viability according to the manufacturer’s instructions.

### AO/PI double staining

Panc1 cells were seeded at a density of 1 × 10^4^ cells. mL^−1^ onto coverslips placed in 24-well plates, following the previously described treatment protocol. After treatment, cells were harvested and washed three times with 1x PBS (D8537, Sigma). Subsequently, the cells were stained with a mixture of Acridine orange and propidium iodide (AO/PI) at 10 μg/mL (AO: 300910250, Thermo Fisher Scientific; PI: P4864, Sigma) in the dark for 5 min at 37 °C. The coverslips were then washed three times with 1x PBS, and fluorescence images were captured at 200x magnification in three representative fields using fluorescence microscopy (Olympus BX63).

### Flow cytometry

The application of flow cytometry was to identify and distinguish cell populations. Panc1 cells were detached using 0.05% trypsin and 0.02% EDTA for 5 min at 37 °C, and the cells were resuspended with 2% FBS in 1x PBS to gain a single-cell suspension. 2 × 10^5^ cells were stained with Annexin V-FITC (640922, BioLegend) and PI in the dark for 30 min at RT. The samples were analysed through an Intellcyt iQue3 flow cytometer (Sartorius) with emission filters of 530 nm for FITC (green) and 615 nm for PI (red). The final step of analyses was performed using iQueForecyt software.

### Migration assay

The migration assay was proceeded using 24-well plate Transwell inserted with an 8 µm pore polyethylene terephthalate membrane (83.3932.800, Sarstedt). Treated cells were detached using 0.05% trypsin and 0.02% EDTA, resuspended in serum-free medium, and centrifuged. Subsequently, 1 × 10^5^ Panc1 cells were resuspended in 100 µL serum-free medium prior to being seeded into the upper chambers. The upper chamber was positioned in a 24-well plate containing 700 µL DMEM supplemented with 10% FBS and the same treatments (control, 10 µM BD B10, 0.1 µM gemcitabine, and co-administration of 0.1 µM gemcitabine with 10 µM BD B10) for incubation over 24 h to continuously exposed to the treatment conditions throughout the migration process. After incubation, the chambers were fixed with 4% formaldehyde at room temperature for 15 min, then stained with 1% crystal violet (U5265, Sigma) for 20 min. Non-migrated cells on the upper membrane surface were removed using cotton swabs. Migrated cells were visualised and imaged in four representative fields per chamber using bright-field microscopy (Olympus BX63).

### Immunofluorescence staining

Panc1 cells were seeded at a density of 1 × 10^4^ cells. mL^−1^ on coverslips. After the treatments, the coverslips were fixed with 4% formaldehyde for 10 min at 37 °C, permeabilised with 0.1% TrixonX-100 for 10 min at RT, and blocked with 5% BSA for 30 min. The coverslips were then incubated with primary anti-Vimentin, anti-N-cadherin, or anti-ß-catenin overnight at 4 °C. The secondary antibody (Alexa Fluor 488 donkey anti-rabbit antibody, R37118, Invitrogen) and DAPI (MBD0015, Sigma) were added and incubated with the coverslips in the dark for 45 min at room temperature. The cells were imaged using fluorescence microscopy (Olympus BX63). The fluorescence intensity of the green channel (per pixel intensity) was quantified and normalised to DAPI (per pixel intensity) using ImageJ. The channels were first separated into blue and green, and the intensity of each was measured individually. Although minor variations in DAPI intensity may occur between treatments, we consider normalisation to DAPI a reliable method for ensuring accurate comparisons across samples.

### RNA-seq and RT-qPCR

Treated Panc1 cells were extracted using the RNeasy Plus Mini Kit (74136, Qiagen) according to the manufacturer’s instructions. RNA concentration and quality were examined using NanoDrop ND 1000 spectrophotometer.

For RNA-Seq data, 1 μg of total RNA was used to perform RNA-Seq analysis. Library preparation and sequencing were performed using an Illumina NovaSeq6000 system (Illumina, San Diego, CA) S1-100 flow cell and 75 bp paired-end reads. The mapping rate to the genome is 81.2%–89.5%, and read depth is 36–45 million reads for all the treatment groups.

Ingenuity pathway analysis (IPA, Qiagen) was used to assess how differential gene expression between gemcitabine alone and co-administration of gemcitabine and BD B10. Differentially expressed genes (DEGs) were filtered using thresholds of  | log2FC | >0.5 and false discovery rate (FDR) < 0.05 (two-tailed testing). Statistically significant canonical pathways were identified in IPA by filtering for p-values <0.05, and results were visualised as bar charts to highlight pathway activation or inhibition.

For RT-qPCR analysis, the single-strand cDNA synthesis was proceeded using the High-Capacity cDNA Reverse Transcription Kit (4368814, Applied Biosystems). RT-qPCR was performed using the LightCycler 480 SYBR Green I Master (04707516001, Roche) in a LightCycler 480 Instrument II (Roche). Primers were designed to span exon-exon junctions to ensure mRNA specificity. Sequences of all the primers are shown below.

**Table:** 

Name	Forward sequence (5'→ 3′)	Reverse sequence (5'→ 3′)
*ACTB*	ATTGGCAATGAGCGGTTC	GGA​TGC​CAC​AGG​ACT​CCA​T
*SLC29A1*	GCT​CAC​TCC​AAA​GTC​TCA​GCA	GGT​GAT​GGT​GTT​CTC​GGT​TT
*ABCB1*	TTG​CTG​CTT​ACA​TTC​AGG​TTT​CA	AGC​CTA​TCT​CCT​GTC​GCA​TTA
*ABCC1*	CTC​TAT​CTC​TCC​CGA​CAT​GAC​C	AGC​AGA​CGA​TCC​ACA​GCA​AAA
*ABCC3*	GAAGCTGGAGCCGAAGGT	AAAAGCAGACGGCACGAC
*ABCG2*	ACG​AAC​GGA​TTA​ACA​GGG​TCA	CTC​CAG​ACA​CAC​CAC​GGA​T
*RRM1*	AGT​TAT​AGA​GGT​CTT​CCA​TCA​CAT​CA	AAG​CAC​CCT​GAC​TAT​GCT​ATC​C
*PIK3CA*	TCT​CCG​TCC​TCG​GAT​TCT​CT	TTC​TTG​TTC​CTC​CTC​AGA​GTC​G
*Nrf2*	AAA​CCA​GTG​GAT​CTG​CCA​AC	TCT​ACA​AAC​GGG​AAT​GTC​TGC
*MYC*	TCT​CCG​TCC​TCG​GAT​TCT​CT	TTC​TTG​TTC​CTC​CTC​AGA​GTC​G
*CTNNB1*	CCT​GTT​CCC​CTG​AGG​GTA​TTT	GCT​CCA​GAA​GCA​GTC​ATC​CA
*FZD5*	GCA​CAA​CCA​CAT​CCA​CTA​CG	GCC​ATG​CCG​AAG​AAG​TAG​AC
*CREB1*	TGA​ACG​AAA​GCA​GTG​ACG​GA	CTG​CTG​GCA​TAG​ATA​CCT​GGG
*VEGF-A*	GAA​GTG​GTG​AAG​TTC​ATG​GAT​GTC	CGA​TCG​TTC​TGT​ATC​AGT​CTT​TCC
*Bcl2*	CCC​GCG​ACT​CCT​GAT​TCA​TT	AGT​CTA​CTT​CCT​CTG​TGA​TGT​TGT
*AIF*	TAC​CTC​AGC​AAC​TGG​ACC​ATG​GAA	TAC​CTT​CCT​GCC​GTC​TTT​CAG​CTT
*E-cadherin*	ATT​GCA​AAT​TCC​TGC​CAT​TC	GTTGTCCCGGGTGTCATC
*N-cadherin*	GTA​TCC​GGT​CCG​ATC​TGC​A	ATA​GTC​CTG​CTC​ACC​ACC​AC
*Vimentin*	AGG​CAA​AGC​AGG​AGT​CCA​CTG​A	ATC​TGG​CGT​TCC​AGG​GAC​TCA​T
*KEAP1*	ATC​GAT​GGC​CAC​ATC​TAT​G	GAT​CCT​TCG​TGT​CAG​CAT​TG
*HO-1*	CCT​CCC​TGT​ACC​ACA​TCT​ATG​T	GCT​CTT​CTG​GGA​AGT​AGA​CAG
*NQO1*	CATCCCAACTGACAACCA	GAA​GCC​TGG​AAA​GAT​ACC​C

### Western blot analysis

Cell lysates were extracted from treated Panc1 cells using RIPA buffer (R0278, Sigma) containing phosphatase inhibitor (K1015, APE x BIO) and protease inhibitor (K1019, APE x BIO). After incubating on ice for 30 min, the cell lysates were centrifuged at 13,000 g for 30 min at 4 °C. Protein concentrations were measured using Pierce BCA Protein Assay Kit (23227, Thermo Fisher Scientific). 40–60 µg proteins were mixed with 4x Laemmli Sample Buffer (161-0747, Bio-Rad) and heated at 100 °C for 8 min to create the samples ready for gel analysis. The samples were separated by SDS-PAGE and electro-transferred onto the polyvinyl difluoride membranes (PVDF, 88518, Thermo Fisher Scientific). The membranes were blocked with 5% milk in 1x TBST for 1 h at room temperature, and then further incubated with the following primary antibodies at 4 °C overnight: anti-E-Cadherin (14472, Cell Signaling Technology); N-Cadherin (13116, Cell Signaling Technology); ß-catenin (8480, Cell Signaling Technology); Vimentin (5741, Cell Signaling Technology); Phospho-Akt-Ser473 (9271, Cell Signaling Technology); Akt (pan) (4691, Cell Signaling Technology); Phospho-STAT3-Tyr705 (9145, Cell Signaling Technology); STAT3 (9139, Cell Signaling Technology); and GAPDH (GTX100118, Genetex). Anti-Rabbit IgG HRP-linked antibody (7074P2, Cell Signaling Technology) and Anti-mouse IgG (A9044, Sigma) were then applied to the PVDF membrane for 1 h at RT, and the band detection was visualised using Pierce ECL Western blotting Substrate (32106, Thermo Fisher Scientific) and the Molecular Imager Gel ChemiDoc™ XRS system (Bio-Rad). Protein band intensities were quantified using ImageJ, with normalisation performed against GAPDH as the internal control.

### Statistical analysis

All data were analysed as mean ± SEM using GraphPad Prism. Comparisons among multiple treatment groups were assessed using one-way ANOVA with Tukey correction. A p value < 0.05 (*p < 0.05; **p < 0.01; ***p < 0.001, ****p < 0.0001) was considered significant.

## Results

### Co-administration of drug sensitiser BD B10 with gemcitabine reduced the cell viability specifically in Panc1 cells

A Tanimoto coefficient analysis helped to show that drug sensitiser BD B10 was structurally similar to trigonelline (Table 1), a PI3K/Akt inhibitor that functions through blocking Nrf2-dependent Bcl2 expression, leading to the activation of the apoptosis pathway which could combat the cell viability of pancreatic cancer cells by maintaining PI3K/Akt inhibition when co-administered with chemotherapy drugs (Nugrahini et al., 2020).

**TABLE 1:** Comparison between Akt inhibitors and BD B10 by Tanimoto coefficient analysis.

Akt inhibitor	Trigonelline	SC66	Honokiol	Loureirin A	ISC-4
Maximum common structure (MCS)	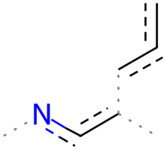	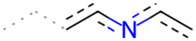	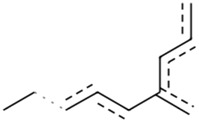	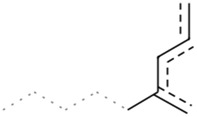	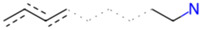
Akt inhibitor (MCS highlight)	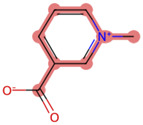	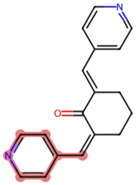	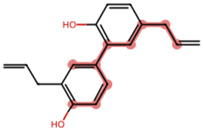	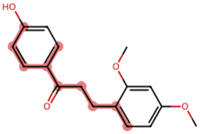	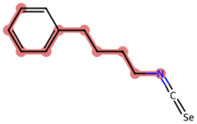
BD B10 (MCS highlight)	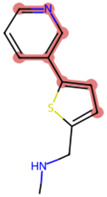	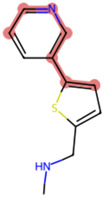	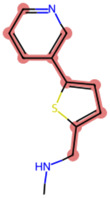	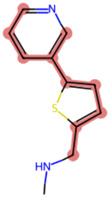	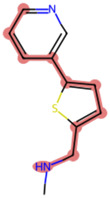
Tc ratio	0.61	0.55	0.54	0.53	0.51

**FIGURE 1:**
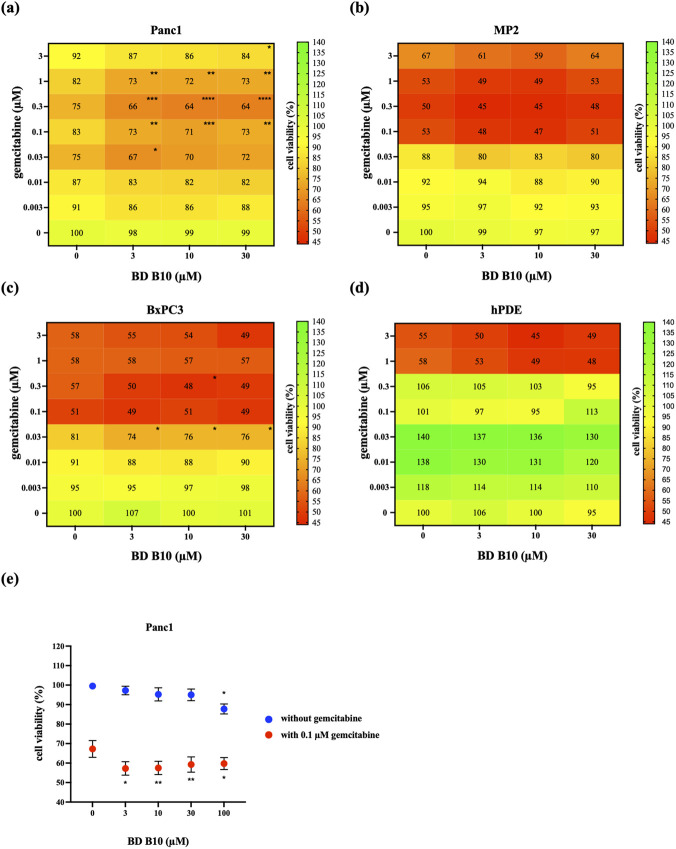
*In vitro* cytotoxic effects of gemcitabine and BD B10 in established pancreatic cell lines. The cell viability of **(a)** Panc1 (n = 7); **(b)** MP2 (n = 2); **(c)** BxPC3 (n = 2); **(d)** hPDE (n = 4) were pre-treated with different concentrations of BD B10 for 24 h, then a further 72 (MP2 and BxPC3) or 96 h incubation of various combinations of gemcitabine and BD B10 were performed. The cell viability assay was analysed using the WST-1 assay at the end of the treatment. Samples with the same concentration of gemcitabine were grouped, and the statistical significance was calculated by comparing to samples without BDs. **(e)** The MTT viability assay was performed on Panc1 cells after incubation with 0.1 µM gemcitabine and 10 µM BD B10 for 96 h (n = 4). Samples with/without gemcitabine were grouped, and the statistical significance was calculated by comparing to samples without BDs. The viable activity of the control (without gemcitabine and BDs) was designated as 100% viability, as shown in each cell line. Data were analysed using GraphPad Prism, with results expressed as mean ± SEM. Comparisons between treatment groups were evaluated using one-way ANOVA to determine statistical significance (*p < 0.05; **p < 0.01; ***p < 0.001, ****p < 0.0001).

We successfully established an increased killing phenotype specific to pancreatic cancer cells by applying the WST-1 cell viability assay after the co-administration of gemcitabine and BD B10 at different concentrations (Figure 1). Pancreatic cancer cells: (1) Panc1, (2) MP2, (3) BxPC3, and (4) the non-cancerous pancreatic cells hPDE were tested. In cancer research, cell viability starting point is typically set between 60% and 80% to provide a good balance between maintaining a healthy cell population and allowing a window to observe treatment effects on cell viability (Ayuda-Du et al., 2023). Gemcitabine exerts its cytotoxicity by inhibiting DNA synthesis, primarily through direct incorporation into replicating DNA and subsequent termination of chain elongation (de Sousa Cavalcante and Monteiro, 2014).

BD B10 is non-toxic to cells in normal conditions, it only showed its cytotoxic impact under co-administration with gemcitabine. Furthermore, using 10 µM BD B10 and 0.1 µM gemcitabine decreased the cell viability ratio by 12% in Panc1 cells (Figure 1a), which showed a statistical significance compared to the group treated with 0.1 µM gemcitabine alone (p < 0.001). While MP2 cells (Figure 1b) showed only an 8% decrease at 3 µM or 30 µM BD B10 with 0.03 µM gemcitabine, without statistical significance. BxPC3 cells (Figure 1c) showed a 7% decrease in cell viability applying 0.03 µM gemcitabine and 3 µM BD B10 when compared to 0.03 µM gemcitabine alone, with statistical significance (p < 0.05). In contrast, the non-cancerous pancreatic cell line hPDE exhibited no toxicity following co-administration of gemcitabine and BD B10 (Figure 1d). However, treatment with 100 µM BD B10 alone in hPDE cells resulted in a marked reduction in cell viability (32%), which did not meet the selection criteria for inclusion in the cell viability comparison (60%–80%).

The results indicated that the drug-enhancing regulation by BD B10 revealed the best killing efficiency in Panc1 cells. The phenotype was further confirmed with the MTT viability assay in Panc1 cells (Figure 1e). Similar results were observed, showing that the co-administration of 0.1 µM gemcitabine and 10 µM BD B10 significantly decreased cell viability (p < 0.01), consistent with the results from the WST-1 viability assay. Additionally, the co-administration experiments were conducted in duplicates, in accordanc with our previous data (Grixti et al., 2017).

### Co-administration of gemcitabine and BD B10 induced the apoptotic-associated morphology in Panc1 cells

**FIGURE 2:**
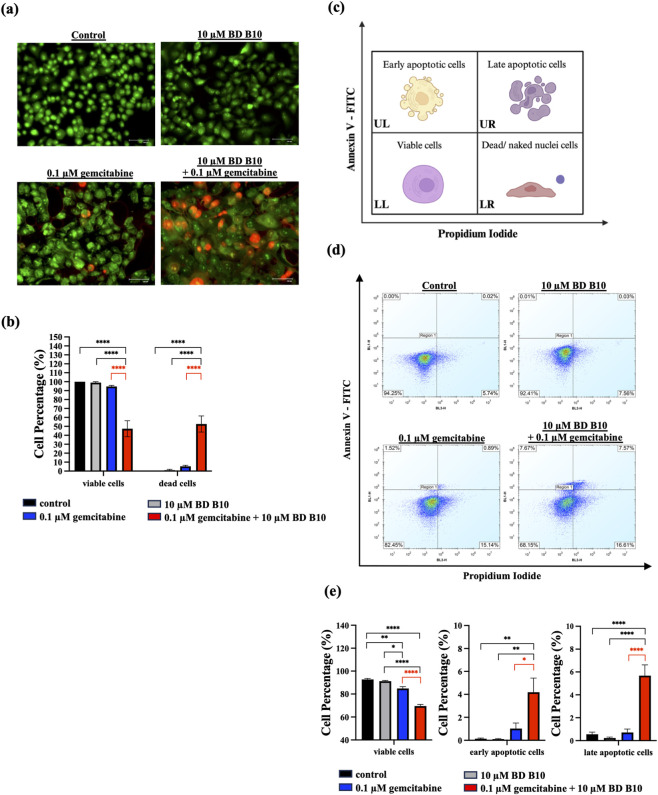
Co-administration of gemcitabine and BD B10 induced apoptosis pathway in Panc1 cells. Apoptosis was analysed using multiple methods: **(a)** AO/PI doubling staining was applied to assess morphological changes in Panc1 cells following each treatment, using fluorescence microscopy, n = 3. Images were captured at 100x magnification (scale bar = 100 µm). **(b)** The proportion of live (green) and dead (red) cells was quantified using ImageJ and normalised to actual cell counts to analyse cell distribution. **(c)** Diagram of different apoptotic stages: viable cells, early apoptotic cells, late apoptotic cells, and dead/naked nuclei cells. **(d)** Annexin V-FITC/PI assay was applied to determine the distribution of different stages of apoptotic cells in Panc1 cells using flow cytometry, n = 4. **(e)** The percentage of each stage of Panc1 cells was statistically quantified by the iQueForecyt software to analyse cell distribution. Data were analysed using GraphPad Prism, with results expressed as mean ± SEM. Comparisons between treatment groups were evaluated using one-way ANOVA to determine statistical significance (*p < 0.05; **p < 0.01; ***p < 0.001, ****p < 0.0001).

To assess the drug-sensitising effect of BD B10, subsequent analyses focused on comparing the co-administration group (gemcitabine plus BD B10) with the gemcitabine alone group, highlighted in red. BD B10 alone and the untreated control served as baseline references. Apoptosis induction was evaluated across the four treatment groups using AO/PI double staining and fluorescence microscopy. Acridine orange, a membrane-permeable green fluorescent nucleic acid dye, labels all cells (viable and dead), whereas PI, a membrane-impermeable red/orange dye, selectively stains late apoptotic and dead cells (Chan et al., 2016). The results demonstrated that co-administration of 0.1 µM gemcitabine and 10 µM BD B10 for 96 h significantly increased dead cell populations (p < 0.0001), which aligned with previous findings. This combination also produced a marked reduction in viable cells compared to gemcitabine alone (p < 0.0001) (Figures 2a,b).

Morphological changes were also observed in both the control and 10 µM BD B10 treatment groups, cells exhibited bright green fluorescence and exhibiting viability consistent with the WST-1 and MTT assay results. However, upon application with 0.1 µM gemcitabine, a few cells displayed red nuclear fluorescence, enlarged nuclei, and evidence of cell shrinkage indicative of apoptosis or senescence. Treatment with co-administration of 0.1 µM gemcitabine plus 10 µM BD B10 induced numerous cells with red fluorescence, signifying that the presence of gemcitabine with BD B10 induces additional cell death, thereby enhancing the cytotoxic effect against pancreatic cancer cells.

The apoptotic cell cycle stages were further characterised using the Annexin V-FITC/PI assay coupled with flow cytometry (FC analysis). The upper left (UL), upper right (UR), lower left (LL), and lower right (LR) quadrants correspond to early apoptotic cells, late apoptotic cells, viable cells, and dead/naked nuclei populations, respectively (Figure 2c). Co-administration of gemcitabine and BD B10 resulted in a statistically significant increase in late apoptotic cells compared to gemcitabine alone (p < 0.0001). Moreover, the proportion of viable cells was markedly reduced in the combined treatment group relative to gemcitabine alone (p < 0.0001), as distinguished in Figures 2d,e. Collectively, these findings demonstrate that BD B10 enhances the cytotoxic activity of gemcitabine against pancreatic cancer cells.

### Co-administration of gemcitabine and BD B10 inhibited the epithelial-mesenchymal transition (EMT) to suppress metastasis in Panc1 cells

**FIGURE 3:**
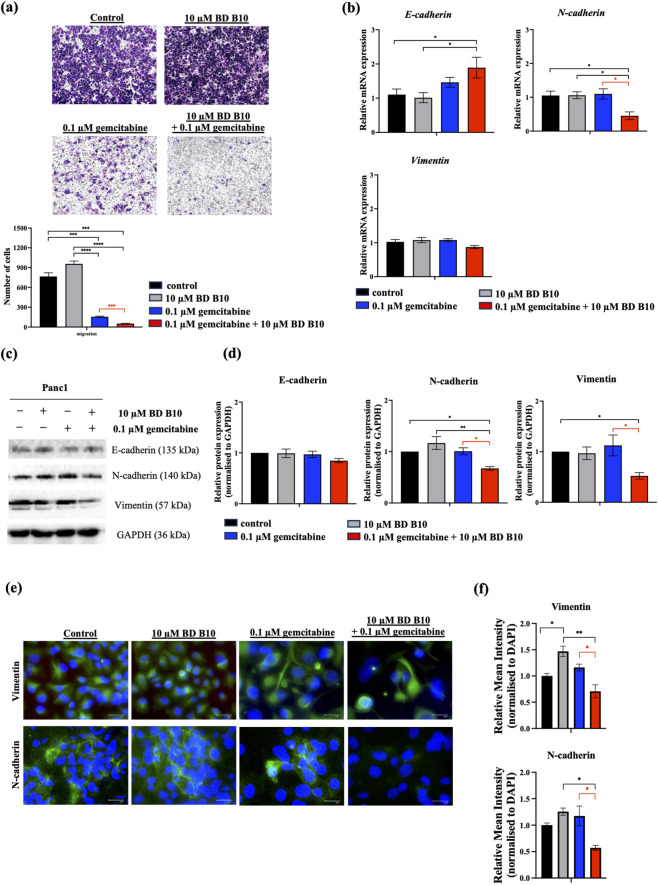
Co-administration of gemcitabine and BD B10 inhibited tumour progression by reducing the epithelial-mesenchymal transition (EMT) in Panc1 cells. EMT progression was analysed using multiple methods: **(a)** Migration assay was performed by seeding treated Panc1 cells in 8 µm transwell chambers for 24 h incubation. Migrated cells were observed and imaged using bright-field microscopy at 100x magnification, n = 3. Additionally, Migrated cells were quantified using ImageJ to perform cell counts in each treatment group. **(b)** The relative mRNA expression of *E-cadherin*, *N-cadherin*, and *vimentin* levels in Panc1 cells were measured by RT-qPCR, n = 10. **(c)** protein expression of E-cadherin, N-cadherin, and vimentin levels in Panc1, n = 4. GAPDH was shown for equivalency of loading. **(d)** The protein quantification of E-cadherin, N-cadherin, and vimentin were made using Image J. **(e)** Immunofluorescence staining of vimentin (n = 3) and N-cadherin (n = 2) were visualised using fluorescence microscopy. Images were captured at 200x magnification (scale bar = 50 µm). **(f)** Fluorescence intensity of vimentin and N-cadherin were quantified and normalised to DAPI using Image J. Data were analysed using GraphPad Prism, with results expressed as mean ± SEM. Comparisons between treatment groups were evaluated using one-way ANOVA to determine statistical significance (*p < 0.05; **p < 0.01; ***p < 0.001, ****p < 0.0001).

The EMT, a hallmark of early pancreatic cancer progression, drives aggressive metastasis and contributes to high mortality rate in pancreatic cancer. BD B10, a PI3K/Akt inhibitor-like drug sensitiser, was evaluated for its potential to suppress the downstream Wnt/ß-catenin cooperating CREB pathway and inhibit metastatic behaviour. We first investigated the metastatic capabilities of each treatment group by using a Transwell-based migration assay (Figure 3a). The control and BD B10 groups exhibited robust metastatic ability, while the application of gemcitabine reduced metastatic capacity by killing cancer cells, though residual cells retained a migratory ability. The co-administration group significantly inhibited migration compared to gemcitabine monotherapy (p < 0.001), suggesting that BD B10 impairs the migration ability of pancreatic cancer cells.

To further investigate the effect of BD B10 on EMT markers, RT-qPCR, Western blot, and immunofluorescence staining were performed in Panc1 cells. Co-administration of 0.1 µM gemcitabine and 10 µM BD B10 significantly suppressed both transcriptional and post-transcriptional expression of N-cadherin compared to gemcitabine alone (p < 0.05). Vimentin, another mesenchymal EMT marker, was also markedly reduced at the post-transcriptional regulation level (p < 0.05) (Figures 3b–d). Immunofluorescence staining confirmed a significant reduction in the expression of both N-cadherin (p < 0.05) and Vimentin (p < 0.05) upon combined treatment (Figures 3e,f). The results suggest that BD B10 inhibits the EMT process in Panc1 cells, thereby potentially limiting tumour progression, escape, and chemoresistance.

### Co-administration of gemcitabine and BD B10 impaired PI3K/Akt and EMT pathways

**FIGURE 4:**
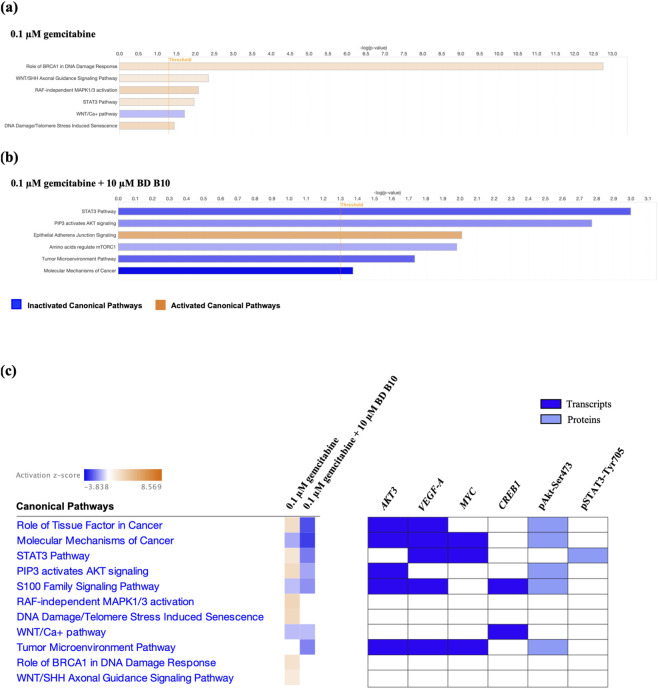
Co-administration of gemcitabine and BD B10 impeded important biological canonical signalling pathways. IPA was utilised to evaluate novel biological pathways from RNA-Seq. Selected individual IPA assessment of **(a)** gemcitabine alone and **(b)** co-administration of gemcitabine and BD B10 identified activation and inactivation canonical signalling pathways in Panc1 cells. **(c)** The comparison analysis investigated biological signalling pathways among co-administration and gemcitabine alone groups. Alongside displayed the candidates selected by canonical signalling pathways involved in co-administration of gemcitabine and BD B10.

To determine the regulatory mechanism of BD B10 in enhancing gemcitabine efficacy, we conducted RNA-Seq to investigate the biological function of BD B10 and its potential downstream targets, combating chemoresistance. IPA was applied to assess how RNA-Seq distinguish differential gene expression between co-administration and gemcitabine alone (Figures 4a–c; Supplementary Tables S1, S2).

The IPA individual and comparison analyses revealed essential tumorigenesis and metastasis downregulation, including STAT3, PI3K/Akt, and Wnt pathways (Supplementary Table S3), while increasing epithelial adherens junction preserved epithelial integrity and led to EMT inhibition. The candidates identified by IPA canonical pathways (Figure 4c), appeared multiple times among the 11 canonical pathways, were selected to confirm the dysregulated pathways affected by BD B10. Amongst the most dysregulated candidates, the transcripts were: *PIK3CA* (an upstream activator of *AKT3*), *VEGFA*, *MYC*, and *CREB1* as identified by RT-qPCR, and the proteins were: pSTAT3-Tyr705 and pAkt-Ser473 following by Western blot analysis.

### Co-administrating gemcitabine and BD B10 inhibited the tumorigenesis and metastasis pathways associated with PI3K/Akt and Wnt/ß-catenin cooperating CREB pathways

**FIGURE 5:**
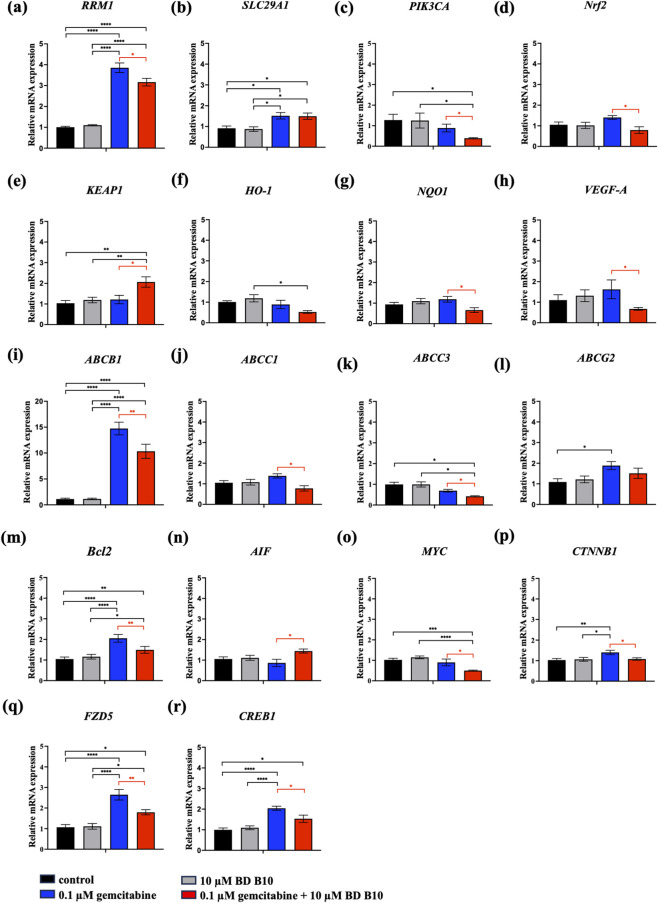
BD B10 enhanced gemcitabine chemosensitivity by inhibiting PI3K/Akt/Nrf2 pathway, Wnt/ß-catenin cooperating CREB pathway, and the expression of multi-drug resistance proteins in Panc1 cells at transcriptional regulation. mRNA expression analyses were performed to investigate the effects of each treatment by RT-qPCR, n = 10. **(a)** Chemoresistance gene (*RRM1*), **(b)** gemcitabine influx transporter (*SLC29A1*), **(c)** PI3K/Akt pathway (*PIK3CA*), **(d**–**g)** Nrf2 pathway (*Nrf2*, *KEAP1*, *HO-1*, *NQO1*), **(h)** angiogenesis (*VEGF-A*), **(I–l)** multidrug resistance efflux transporters (*ABCB1*, *ABCC1*, *ABCC3*, *ABCG2*), **(m,n)** cell apoptosis (*Bcl2*, *AIF*), **(o)** tumorigenesis (*MYC*), and **(p-r)** Wnt/ß-catenin cooperating CREB pathway (*CTNNB1*, *FZD5, CREB1*). Data were analysed using GraphPad Prism, with results expressed as mean ± SEM. Comparisons between treatment groups were evaluated using one-way ANOVA to determine statistical significance (*p < 0.05; **p < 0.01; ***p < 0.001, ****p < 0.0001).

**FIGURE 6:**
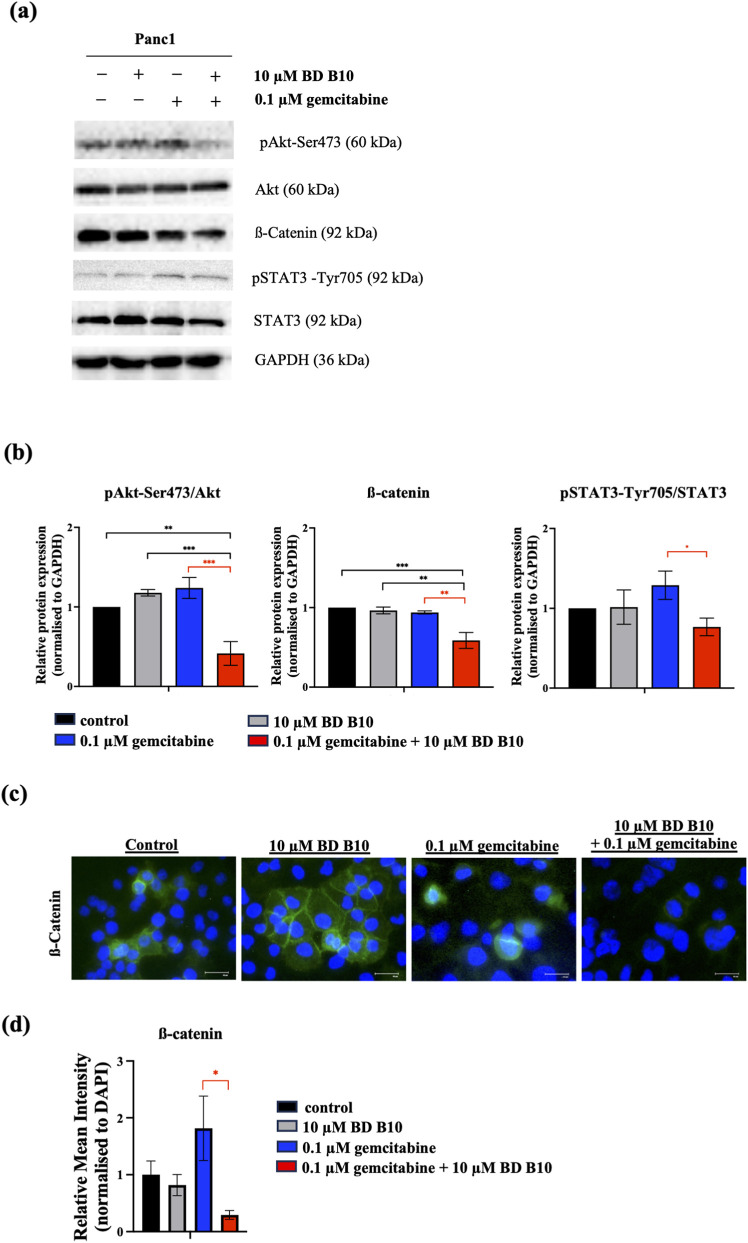
BD B10 inhibited pAkt-Ser473, pSTAT3-Tyr705, and ß-catenin in Panc1 cells at post-transcriptional regulation. **(a)** Protein expression of pAkt-Ser473, Akt, ß-catenin, pSTAT3-Tyr705, STAT3 levels in Panc1 cells, n = 4. GAPDH was shown for equivalency of loading. **(b)** The quantification of each protein band was made using Image J. **(c)** Immunofluorescence staining of ß-catenin was performed and visualised using fluorescence microscopy, n = 3. Images were captured at 200x magnification (scale bar = 50 µm). **(d)** Fluorescence intensity of ß-catenin was quantified and normalised to DAPI using Image J. Data of both Western blot and Immunofluorescence staining were analysed using GraphPad Prism, with results expressed as mean ± SEM. Comparisons between treatment groups were evaluated using one-way ANOVA to determine statistical significance (*p < 0.05; **p < 0.01; ***p < 0.001, ****p < 0.0001).

Regulatory mechanisms were further investigated using RT-qPCR and Western blot analyses (Figures 5, 6; Supplementary Figure S1). The *RRM1* gene, encoding ribonucleotide reductase, is an established molecular target involved in gemcitabine resistance (Bepler et al., 2006). The RT-qPCR data showed increased expression of *RRM1* following gemcitabine treatment alone. However, co-administration of gemcitabine and BD B10 significantly restored gemcitabine sensitivity, as indicated by a marked reduction in *RRM1* expression compared to gemcitabine alone (p < 0.05) (Figure 5a). Additionally, BD B10 exerted inhibitory effects on the PI3K/Akt pathway at both the mRNA (p < 0.05) and phosphorylated protein levels (p < 0.001) (Figures 5c, 6a,b). The downstream effector ß-catenin, a key component of the Wnt/ß-catenin cooperating CREB signalling pathway implicated in metastasis and cancer recurrence (Katoh, 2017), also displayed significant decreases in mRNA (p < 0.05) and protein expression (p < 0.01) relative to gemcitabine alone (Figures 5p–r; Figure 6a,b). Immunofluorescence staining further confirmed a significant reduction in ß-catenin expression upon combined treatment (p < 0.05) (Figures 6c,d). Furthermore, a PI3K downstream target STAT3, which was also selected from the RNA-Seq data, its phosphorylation form was significantly suppressed (p < 0.05) (Figures 6a,b), alongside inhibition of the downstream oncogene *MYC* (p < 0.05) (Figure 5o). These data indicate that BD B10 attenuates oncogenic signalling, tumour survival, and cancer metabolism in Panc1 cells.

### BD B10 triggered drug sensitivity by inhibiting the Ros/Akt/Nrf2 pathway

Early drug resistance is associated with continuous gemcitabine administration, which increases reactive oxygen species (ROS) production and activates the Akt/Nrf2 pathway. This activation leads to the upregulation of multidrug resistance proteins, including ABCB1, ABCC family members, and ABCG2, which function as efflux pumps to expel chemotherapeutic agents from cancer cells. BD B10 demonstrated its sensitising effect under co-administration, particularly when ROS levels were elevated due to gemcitabine exposure. BD B10 significantly reduced activation of the PI3K/Akt/Nrf2 pathway, which operates downstream of ROS and is implicated in tumorigenesis and chemoresistance, and it also downregulated the mRNA expression of (1) efflux transporters *ABCB1* (p < 0.01) (Figure 5i), *ABCC1* (p < 0.05) (Figure 5j), and *ABCC3* (p < 0.05) (Figure 5k) significantly, and (2) angiogenesis fact *VEGF-A* (p < 0.05) (Figure 5h), thereby resuscitating gemcitabine sensitivity in Panc1 cells. Importantly, *SLC29A1* (ENT1) is the primary influx transporter medicating gemcitabine uptake, and BD B10 did not affect gemcitabine import via this regulation (Figure 5b).

### BD B10 increased activation of the apoptosis pathway by inducing apoptosis-inducing factor (AIF) triggering Bcl2 expression in Panc1

The pro-apoptotic effects of BD B10 were demonstrated by flow cytometry and AO/PI double staining assays. Additionally, analysis of the associated signalling pathways revealed that co-administration of gemcitabine and BD B10 significantly suppressed Bcl2 mRNA expression (Figure 5m) by inhibiting the Akt/Nrf2 downstream signalling pathway (Figures 5d–g). Downregulation of Bcl2 was accompanied by a statistically significant increase in AIF release from mitochondria, promoting cell apoptosis (p < 0.05) (Figure 5n).

### The cellular uptake of BD B10 was investigated by comparing the natural metabolites library by Tc

**FIGURE 7:**
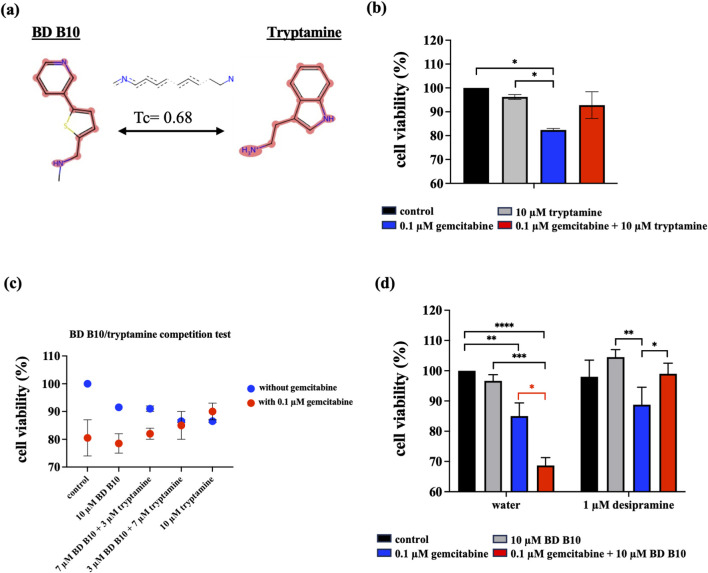
BD B10 uses 5-HTR as a major delivery system to diminish tumour cell growth. **(a)** The most similar natural metabolite to BD B10 was identified as tryptamine by comparing BD B10 with the natural metabolite library using Tc. WST-1 cell viability was performed on the following experiments: **(b)** Treatments with/without tryptamine in Panc1 cells, n = 2. **(c)** The tryptamine/BD B10 receptor competition test, n = 2. **(d)** Treatments with/without SERT inhibitor desipramine, n = 5. Data were analysed using GraphPad Prism, with results expressed as mean ± SEM. Comparisons between treatment groups were evaluated using one-way ANOVA to determine statistical significance (*p < 0.05; **p < 0.01; ***p < 0.001, ****p < 0.0001).

To elucidate the mechanism by which BD B10 enhances gemcitabine sensitivity in pancreatic cancer, we compared the fingerprint-based structural similarity between BD B10 and compounds in a natural metabolites library using Tc analysis (Table 2). Since the uptake mechanisms of most natural metabolites are well-characterised, structurally similar candidates may be up-taken via analogous transport systems. As shown in Figure 7a, tryptamine exhibited notable similarity to BD B10 (Tc: 0.68), which is primarily taken up through the 5-hydroxytryptamine receptor (5-HTR).

**TABLE 2:** Comparison between BD B10 and natural metabolites by Tanimoto coefficient analysis.

Natural metabolite	tryptamine	N-Methylserotonin	Serotonin (1+)	Metanephrine	Indol-3-ylacetaldehyde
Maximum common structure (MCS)	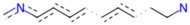	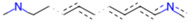	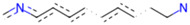	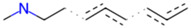	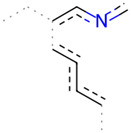
BD B10 (MCS highlight)	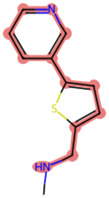	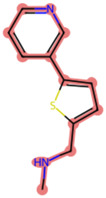	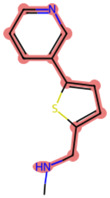	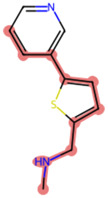	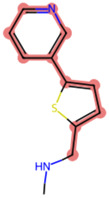
Natural metabolite (MCS highlight)	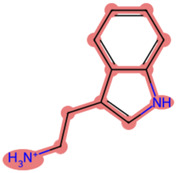	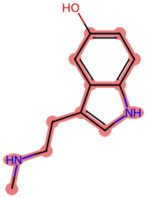	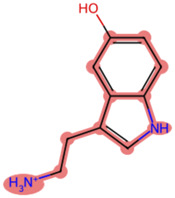	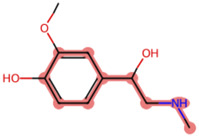	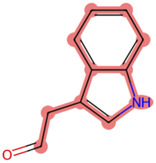
Tc ratio	0.68	0.67	0.65	0.65	0.65

Natural metabolite	Formyl-5-hydroxykynurenamine	3-Methoxytyramine	1-Methylnicotinamide	Epinephrine	Indole-3-acetate
Maximum common structure (MCS)	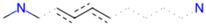	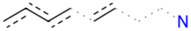	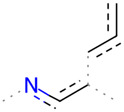	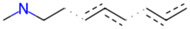	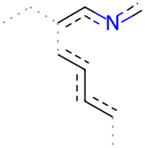
BD B10 (MCS highlight)	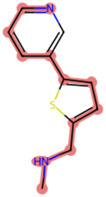	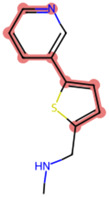	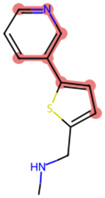	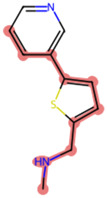	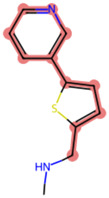
Natural metabolite (MCS highlight)	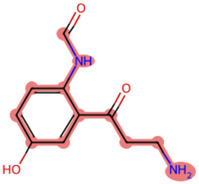	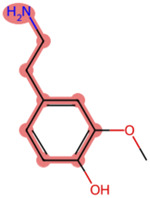	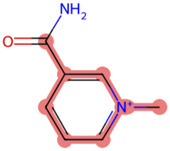	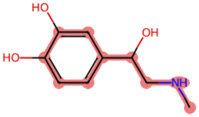	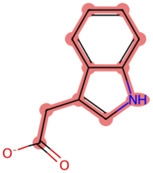
Tc ratio	0.65	0.64	0.64	0.64	0.63

### BD B10 was mainly up-taken by 5-HTR, inhibiting the tumorigenesis pathways in Panc1 cells

Tryptamine is a naturally occurring compound that serves as the structural backbone for various psychoactive substances, including serotonin (5-HT) and psychedelic tryptamines. These compounds primarily exert their hallucinogenic or psychedelic effects through activation of 5-HTRs (Araujo et al., 2015). Activation of 5-HTRs by ligands such as 5-HT and tryptamine has been determined to induce the PI3K/Akt pathway, thereby generating the anti-inflammatory microenvironment that facilitates tumour immune evasion (Karmakar and Lal, 2021). Figure 7b illustrated the cell viability results of co-administration of gemcitabine and tryptamine in Panc1 cells. Unlike the enhanced cytotoxic impact from BD B10 as shown in Figures 1a,e, tryptamine promoted tumour cell survival by enhancing 5-HTR activity. We hypothesise that the structural similarity between BD B10 and tryptamine enabled a receptor binding competition to 5-HTR. In BD B10/tryptamine competition assay (Figure 7c), increasing concentrations of either BD B10 or tryptamine led to antagonistic results in Panc1 cell viability, indicating that 5-HTR serves as a principal receptor for both BD B10 and tryptamine, thereby modulating their downstream effects.

From previous data, BD B10 was seen to mainly use 5-HTR to initiate downstream inhibition in Panc1 cells. However, direct inhibition of SERT would provide strong evidence to confirm how BD B10 delivers its inhibitory message to the Akt pathway and enhances the cytotoxicity specific to pancreatic cancer.

Desipramine hydrochloride, a common SERT inhibitor, was used to block serotonin uptake/reuptake in the cytoplasm. To identify the uptake regulation of BD B10 with SERT, 1 µM desipramine hydrochloride was added across 4 treatments. In the co-administration protocol, 1 µM desipramine hydrochloride was introduced alongside gemcitabine and BD B10. The results (Figure 7d) demonstrated that inhibition of SERT abrogated the enhanced cytotoxic effect of BD B10, such that cell viability was no longer reduced beyond that observed with gemcitabine treatment alone. The findings provide additional evidence that the efficacy of BD B10 is dependent on SERT-mediated uptake in Panc1 cells.

## Discussion

Multidrug resistance significantly impacts the outcome and effectiveness of chemotherapeutic drugs combating PDAC, posing a major challenge in developing new drugs and therapies. Therefore, targeting molecular pathways such as the PI3K/Akt/Nrf2 pathway majorly involved in MDR, leading to tumorigenesis, DNA repair, EMT, and drug efflux regulation, has become a primary focus in drug development (Garg et al., 2024).

During the early stage of drug development and discovery the pharmaceutical industry typically relies on high-throughput screening (HTS) assays of large libraries often comprised from chemical “hit” compounds. Nevertheless, this approach is often time-consuming, pricey, and inefficient (Blanco-Gonza et al., 2023). Despite the development of new methods like phenotypic drug discovery (PDD) and target-based strategies over the past decades, substantial challenges such as hit validation and target deconvolution have remained unresolved (Moffat et al., 2017). An estimation in 2023 indicates that only 1 in 10,000 chemical candidates successfully develop into FDA-approved drugs, with an average development time of 7–10 years (Hardman et al., 2023). Therefore, there is capacity for more effective methods for screening and drug development, and where therapeutic index can be applied as a measure of drug toxicity used to improve drug efficacy by reducing drug dosage (Chen Y. S. et al., 2024; Manzari et al., 2021).

This study suggests that BD B10 acts as a drug sensitiser (Chen Y. S. et al., 2024), increasing drug efficacy when co-administrated with gemcitabine by enhancing cytotoxicity specifically in pancreatic cancer cells, resulting in an additional 12% killing compared with the gemcitabine-alone group. Furthermore, the presence of BD B10 allows for a 10% reduction of gemcitabine dosage while maintaining the same therapeutic effect as a sole treatment of gemcitabine (Figure 1).

Among the three PDAC cell lines evaluated, Panc1 cells were selected as the primary model for investigating the enhancement of gemcitabine efficacy by BD B10, as neither MP2 nor BxPC3 cells exhibited a comparable response to BD. However, Panc1 cells are classified as quasi-mesenchymal (low E-cadherin and high vimentin expression), and therefore lie toward the mesenchymal end of the epithelial-mesenchymal spectrum, which limits their suitability for studying EMT initiation (Ungefroren et al., 2022). For future investigation of gemcitabine efficacy by BD in the context of epithelial plasticity, (1) comparing an epithelial cell line (BxPC3) with a mesenchymal cell line (MP2 or Panc1) in parallel, or (2) applying a 3D culture system to mimic the *in vivo* environment may provide a more appropriate MET model than applying Panc1 cell line alone (Shichi et al., 2019; Puls et al., 2017).

BD B10 is non-toxic when used alone and only exhibits its toxicity enhancement when administrated with gemcitabine. Therefore, BD B10 serves as a drug sensitiser that reduces gemcitabine dose and increases related toxicity. While investigating the effects of BD B10 on Panc1, MP2, and hPDE cells, a biphasic or hormesis dose-response effect was observed in the gemcitabine-only treatment group. In this context, for Panc1 and MP2 cells at higher concentrations of gemcitabine, cell viability appeared higher than in low-dose samples, likely due to an adaptive cell response to stress. Similarly, for hPDE cells, this effect was reported in lower concentrations of gemcitabine. This effect was previously reported in our Grixti et al., 2017 publication.

We uncovered the regulatory mechanisms by which BD B10 enhances drug efficacy in pancreatic cancer (Figures 4–7). BD B10 inhibits the expression of Akt and Nrf2, directly affecting the regulation of proteins that compromise multidrug resistance. It is noteworthy that elevated levels of ROS activate Nrf2, and ROS modify the sensor cystines in Keap1, leading to stabilisation, accumulation, and translocation of Nrf2, which induces ROS detoxication. Chemotherapeutic drugs such as gemcitabine exert their effects on killing malignant tumours by elevating high levels of ROS, prompting apoptosis, autophagy, and lysosome activation (Jiang et al., 2023). However, prolonged ROS exposure can also induce the activation of the PI3K/Akt and Wnt/ß-catenin cooperating CREB pathways, leading to early drug resistance, metastasis, and tumour survival.

**FIGURE 8:**
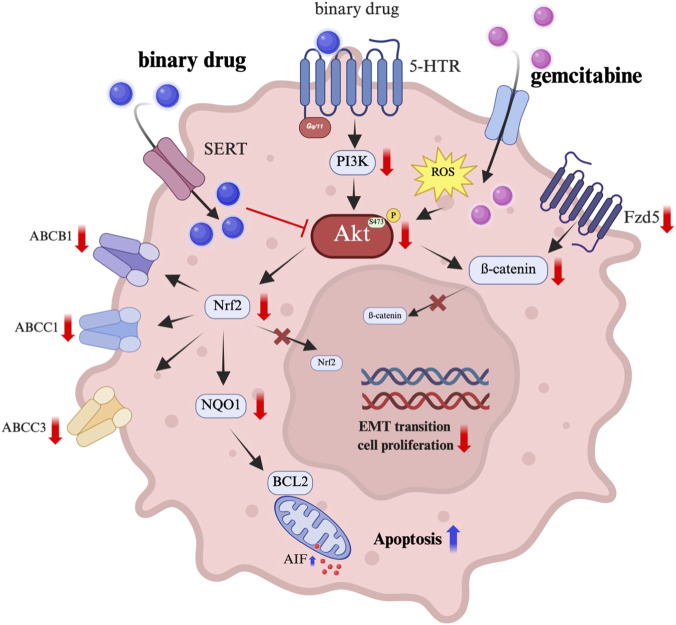
Schematic view of the regulatory mechanism of BD B10 in pancreatic cancer cells. The diagram illustrates key signalling pathways and outcomes of BD B10 in pancreatic cancer. When BD B10 was treated alone, cellular phenotypes were similar to the control, with BD B10 partially interfered with Akt phosphorylation. However, as a small alkaloid molecule, BD B10 was exported by ABC transporters, diminishing its effects on pancreatic cancer cells. Gemcitabine treatment alone led to a continuous increase in ROS levels, which activated the Akt/Nrf2 pathway and promoted rapid development of chemoresistance through upregulation of survival and drug export mechanisms. In contrast, co-administration of gemcitabine and BD B10 significantly inhibited PI3K/Akt phosphorylation, thereby suppressing downstream processes related to drug efflux, angiogenesis, metastasis, and tumour survival in pancreatic cancer cells. This combination effectively counteracted the chemoresistance typically induced by gemcitabine alone.

An advantage of BD B10 lies in its selective enhancement of gemcitabine efficacy during co-administration. Concurrently, BD B10 inhibits ROS-induced Akt activation and reduces the expression of multidrug resistance proteins involved in gemcitabine efflux. Our data demonstrates that BD B10 downregulates the PI3K/Akt/Nrf2 and Wnt/ß-catenin cooperating CREB signalling pathways, as well as key multidrug resistance proteins (ABCB1, ABCC1, ABCC3), increasing cytotoxicity and gemcitabine sensitivity to pancreatic cancer cells (Figure 8).

These investigations have established a new type of sensitiser termed a binary drug (BD), characterised by affording gemcitabine higher specificity in PDAC. This was achieved through an effective and economical Tc-based screening of a low molecular weight chemical fragment library. Current PDAC chemotherapy applies the multi-drug combination FOLFIRINOX as a major second-line chemotherapy for advanced unresectable pancreatic cancer with better overall survival outcomes than gemcitabine monotherapy and NALIRIFOX to treat metastatic PDAC (Urooj et al., 2024). However, severe toxicity and adverse clinical problems remained unsolved (Conroy et al., 2022; Matsumoto et al., 2020; Tezuka et al., 2022). The application of BD decreases the drug dosage while increasing the drug efficacy specific to pancreatic cancer cells, revealing a better potential for gemcitabine to serve as a more effective chemotherapeutic drug for treating advanced pancreatic cancer. Furthermore, we seek to develop cancer-specific binary drugs for medical therapies, advancing personalised medicine applications in the future. This approach which reduces the amount of therapeutic agent has high potential for use in personalised medicine, and may have applications for lowering dose of drugs that failed approval because of adverse effects.

## Data Availability

The RNA-seq data presented in the study have been deposited in the ArrayExpress database at EMBL-EBI under accession number E-MTAB-16607.

## Supplementary material

SUPPLEMENTARY FIGURE S1 Full Western blot analysis used for quantification. Three biological replicates (not shown in the manuscript) of Western blot analysis were presented as representative blots in the main figures.
