# Supplementary figures and images for: Enhancement of gemcitabine toxicity and specificity through PI3K/Akt/Nrf2 pathway inhibition in pancreatic cancer
Source: Front Pharmacol. 2026 Feb 16;17:1724989. doi: 10.3389/fphar.2026.1724989 (PMC12950954; doi:10.3389/fphar.2026.1724989)

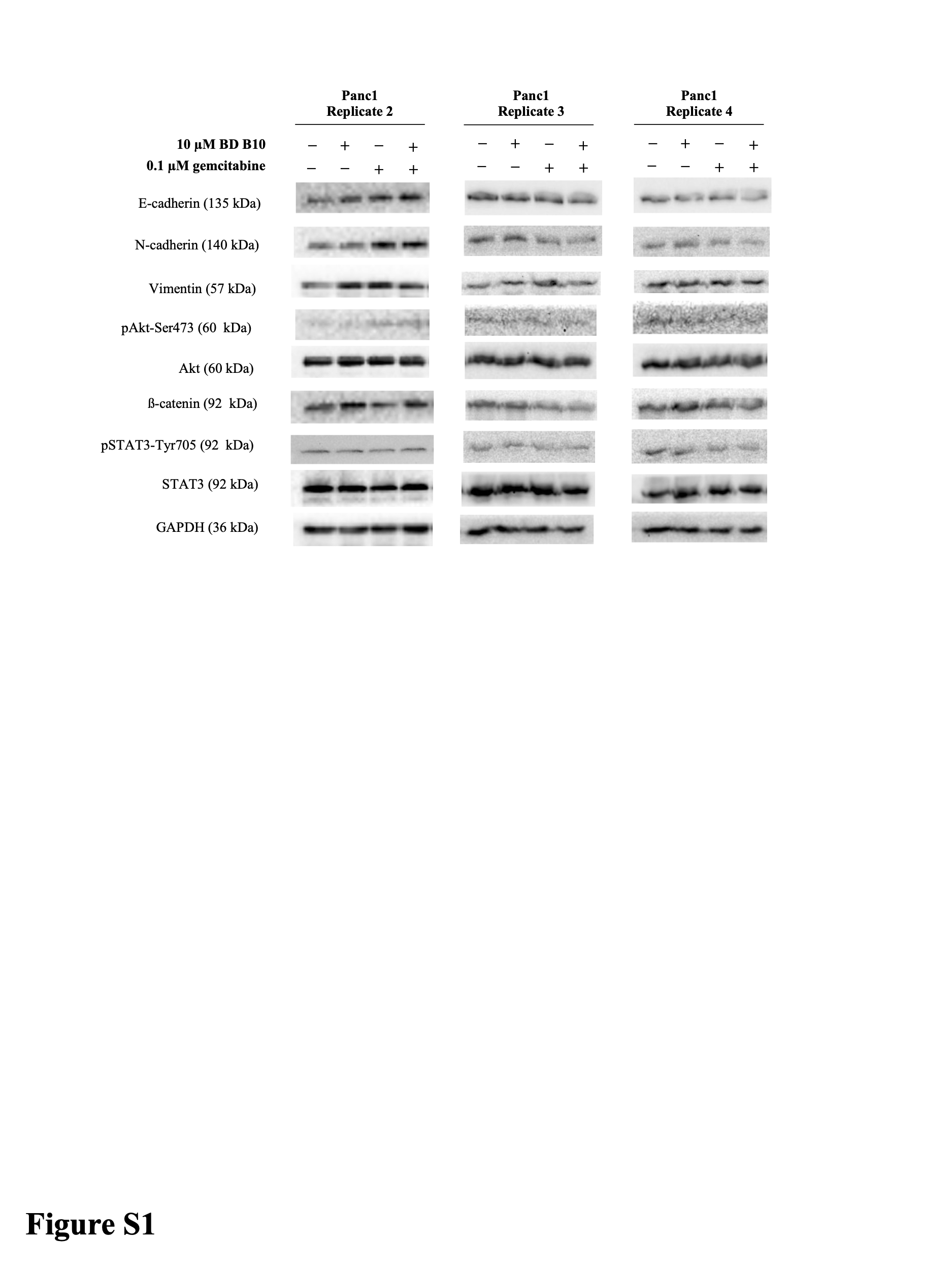

Supplement: Supplementary file 2 [file Image1.tiff]
